# Feasibility study of use of desflurane combined with dexmedetomidine in inhibiting postoperative neurocognitive disorders in elderly patients under general anesthesia: A perspective study

**DOI:** 10.1002/ibra.12073

**Published:** 2022-11-23

**Authors:** Yue Hu, Yong Ye, Hui Lin, Jun‐Jie Chen, Ting‐Ting Sun, Gong‐Wei Zhang, Na Wang, Yuan‐Hang Shu, Xue Gong, Fei‐Fei Ran, Jia‐Li Zhang, Yong Tao

**Affiliations:** ^1^ Department of Anesthesiology The First People's Hospital of Shuangliu District Chengdu Sichuan China; ^2^ Department of Anesthesiology University of Virginia Charlottesville VA United States; ^3^ School of Anesthesiology Southwest Medical University Luzhou Sichuan China

**Keywords:** desflurane, dexmedetomidine, MMSE score, perioperative neurocognitive disorder

## Abstract

This study aimed to explore whether the combined application of desflurane and dexmedetomidine (Dex) reduces the occurrence of postoperative neurocognitive disorders (PND) in patients. We selected patients in our hospital who underwent surgery under general anesthesia, and divided them into two groups: Dex and desflurane (Dex + Des) and desflurane (Des) groups. The data of patients were collected and the Mini‐Mental State Examination (MMSE) score was used to assess cognitive status. The blood cell counts were determined preoperatively and on postoperative days 1, 3, and 6, and the percentage of neutrophils and lymphocytes were also recorded. The statistical methods used were the independent‐samples *t*‐test and the *χ* ^2^ test. Pearson's correlation was used to analyze the correlation between PND and inflammation. The incidence of PND in the Dex + Des group was lower than that in the Des group. The postoperative MMSE scores in the Dex + Des group were higher than those in the Des group (*p* = 0.032). The percentage of neutrophils in the Dex + Des group was significantly lower than that in the Des group on the first and third days after surgery (*p* = 0.007; *p* = 0.028). The MMSE scores on the first day after surgery were negatively correlated with the multiple changes in white blood counts and the percentage of neutrophils (*r* = −0.3038 and −0.3330). Dex combined with Des reduced the incidence of PND and reduced the postoperative inflammatory cell counts.

## Graphical abstract

Patients undergoing general anesthesia in our hospital were divided into desflurane and dexmedetomidine (Dex + Des) group and a desflurane (Des) group. Preoperative and postoperative MMSE scores, percentage of white blood cells, and neutrophil and lymphocyte counts were determined for postoperative exploration of whether the combined use of Des and Dex reduces the occurrence of PND in patients. The results showed that the combined use of Des and Dex could reduce the occurrence of postoperative PND and was negatively correlated with the postoperative inflammatory cell process.

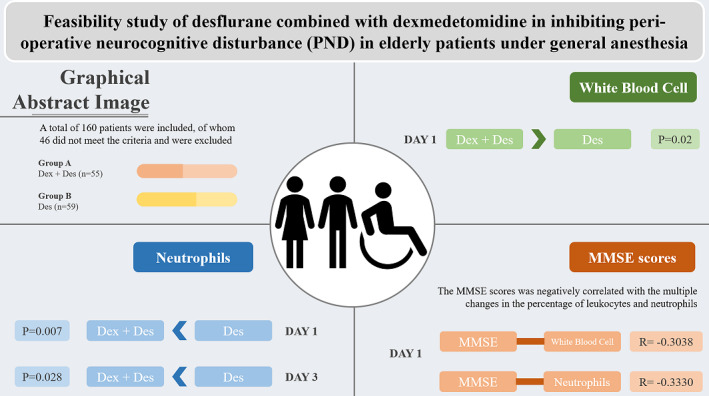

## 1 INTRODUCTION

Whether general anesthesia can affect postoperative cognition remains a controversial topic worldwide; however, there are considerable data involving postoperative cognition among patients after general anesthesia. There are also considerable data showing that there is a delay in cognitive recovery of patients after general anesthesia. It is known that narcotics and surgical trauma have long‐term positive and negative effects on neurocognitive disorders after surgery.

Postoperative neurocognitive disorders (PNDs) describe changes in postoperative cognitive function that can occur after anesthesia or surgery. The concept has been widely recognized and examined since it was proposed in 2018.^1^ As a neuropsychiatric complication, PND affects the rapid recovery of patients during the perioperative period and increases the hospitalization time and treatment cost. The systemic stress response caused by the release of cytokines during anesthesia and surgery may cause changes in brain function and play a role in the development of postoperative cognitive dysfunction.^2^

Desflurane (Des) is a volatile inhaled anesthetic. Studies have shown that Des reduces the loss of nerve function and protects the brain^3^; however, Des also increases the inflammatory response more than propofol.^4^ Indeed, surgical trauma and the use of anesthetic drugs can produce inflammatory responses.^5^ The use of Des to maintain anesthesia during surgery can lead to genotoxicity and inflammatory responses,^6^ which can also lead to brain injury.

It has been shown that dexmedetomidine (Dex) significantly reduces the incidence of emergence delirium in children.^7^ In the elderly undergoing orthopedic surgery, the use of Dex reduces the levels of inflammatory factors and the incidence of postoperative cognitive dysfunction (POCD).^8^ Dex is an effective 2‐adrenaline receptor agonist with clinical indications for sedation or antianxiety, and adverse reactions, including hypotension, bradycardia, sinus arrest, and transient hypertension.^9^

Interestingly, Des was found to have neurotoxic effects on motor neurons, and more importantly, Dex mitigated this process, which may indicate its application in protecting motor neurons from neurotoxic effects. This study provides evidence for the protective effect of Dex on Des‐induced motor neuron death^10^ In a pediatric study, Dex reduced the incidence of sudden irritation, postoperative nausea and vomiting, postoperative pain, and Oculocardiac reflex in patients who had undergone pediatric strabismus surgery.^11^ Compared with the use of propofol + Des and Des + Dex, we found that the postoperative extubation time of Dex was significantly shortened, indicating that the use of Dex + Des can achieve rapid extubation.^12^ A meta‐analysis of perioperative postoperative delirium (POD) in 39 randomized controlled trials and including 5991 patients under general anesthesia showed that Dex may be the most effective sedative to reduce POD.^13^

A severe stress response may be an important cause of PND, while Dex may reduce PND by inhibiting stress and the inflammatory response. Here, we hypothesized that Dex combined with Des anesthesia can reduce stress and inflammatory responses, reduce the occurrence of PND, and improve cognitive function in elderly patients.

## 2 MATERIALS AND METHODS

### 2.1 Ethical statement

The populations included in this study were patients who attended the First People's Hospital of Shuangliu District, Chengdu, and informed consent was obtained from all the patients. This study was a randomized, parallel controlled trial, double‐blind to the subjects and statisticians. The current study complied with the Consolidated Standards of Reporting Trials (CONSORT) guidelines, was conducted between May 2019 and February 2020, and was approved by the Ethics Committee of The First People's Hospital of Shuangliu District, Chengdu (Approval number: 20180101). Similarly, this study was approved by the Chinese Clinical Trial Registry (Clinical Trial Registration Number: ChiCTR1900023385).

### 2.2 Patients and grouping

**Figure 1:**
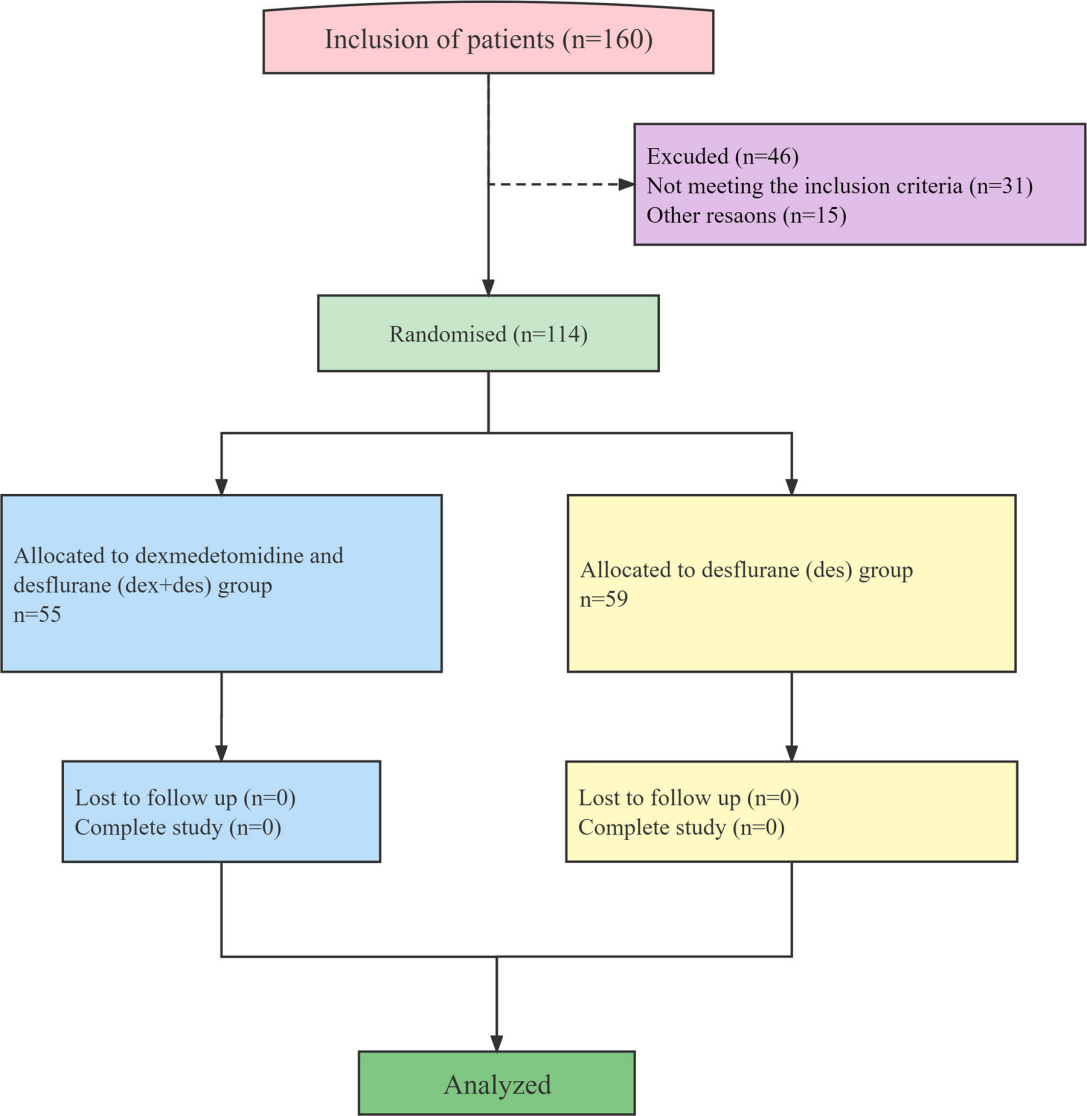
Flow diagram of the patients in this study

A total of 160 patients were included, of whom 46 did not fulfill the criteria and were excluded (Figure 1). All patients who fulfilled the inclusion criteria were randomly divided into the Dex + Des group (*n* = 55) and the Des group (*n* = 59) using Microsoft Excel. [Correction added on 17 May 2024, after first online publication: The word “Des group (*n* = 55) and the Dex + Des group (*n* = 59)” was revised to “Dex + Des group (*n* = 55) and the Des group (*n* = 59)” in the preceding sentence.]

The inclusion criteria for this study were as follows: (1) for patients who had undergone elective general anesthesia for 1–2 h, the endotracheal catheter was removed after surgery; (2) older than 60 years of age; (3) American Society of Anesthesiologists classification level I–II; (4) Mini‐Mental State Examination (MMSE) core > 24; and (5) voluntarily signed informed consent.

The exclusion criteria included the following: (1) presence of allergic or abnormal reaction to Des; (2) presence of allergic or abnormal reaction to Dex; (3) preoperative patients with cognitive dysfunction and hearing impairment; (4) patients with a history of long‐term use of narcotic analgesics, sedatives, and antidepressants; (5) patients with a body mass index (BMI) ≥ 35 kg/m^2^; (6) patients with a preoperative acute infection; (7) patients who could not comply with follow‐up evaluations; (8) patients who had participated in other clinical trials within 3 months before the current study; (9) patients with a history of severe cardiovascular disease (e.g., coronary heart disease, congenital heart disease, and ≥Grade 3 cardiac function); (10) patients with a history of alcohol and drug use; (11) patients with mental disorders; and (12) patients who could not communicate.

### 2.3 Anesthesia protocol

Before anesthesia was administered, peripheral venous access was established and the patient was administered 4 ml/kg/h of equilibrium fluid. A multifunctional monitor was also used to monitor the heart rate (HR), electrocardiogram, systolic blood pressure, pulse oxygen saturation, and end‐expiratory carbon dioxide. Patients were connected to the bispectral index (BIS) upon arrival to the operating theater. Anesthesia was sequentially induced intravenously with sufentanil (0.4 μg/kg) and propofol (2 mg/kg), and cisatracurium (0.3 mg/kg) for endotracheal intubation. Then, mechanical ventilation was initiated. The ventilator parameters were as follows: tidal volume, 8–10 ml/kg; respiratory frequency, 8–12 times/min; respiratory ratio, 1:2; oxygen flow rate, 2 L/min; and end‐expiratory CO_2_ partial pressure, 30–40 mmHg.

In the Des group, anesthesia was maintained with a 3.5%–6% volume fraction of Des. The Dex + Des group was treated with 3.5%–6% volume fraction of Des and Dex (0.5 μg/kg/h). A remifentanil infusion (0.05–2 µg/kg/min) was administered to all groups. Neuromuscular block was monitored using a nerve stimulator. Cisatracurium (0.05–0.08 mg/kg/h) was added intraoperatively and Dex was discontinued 10 min before the operation was completed, followed by an analgesic pump. BIS monitoring was maintained at 40–50. Postoperatively, the fresh air flow was increased to 6 L/min (100% O_2_). The extubation indicators were as follows: respiratory rate, ≥8/min; tidal volume, ≥5 ml/kg; systolic pressure, 80–150 mmHg; cough reflex recovery; train‐of‐four stimulation ≥ 0.9; and SpO_2_ ≥ 97%. After extubation and continuous intravenous pumping, patient‐controlled intravenous analgesia was formulated as follows: 90 ml of 0.9% normal saline; 150 µg of sufentanil; and 5 mg of toanesetron. The analgesic pump was set as follows: 1 ml of single patient‐controlled anesthesia; 10 min of locking time; and 2 ml/h of background dose. The postoperative analgesia was maintained for 2 days. The patient was extubated and transferred to the postanesthesia ward. Hemrheology, including the HR and the mean arterial blood pressure (MABP), was recorded at baseline and every 15 min. The recovery time from anesthesia was recorded using the Aldrete scoring system (time from cessation of all anesthesia to full recovery).

### 2.4 Trial data collection

The patients' demographic information, including sex, age, height, weight, operative time, and anesthesia time, were recorded. The improved Aldrete score^14^ was used to assess recovery of consciousness 6 h postoperatively, and the MMSE score was used to assess cognitive function before the operation and 24 h postoperatively. The MMSE was completed within 15 min. An MMSE score < 26 postoperatively indicates cognitive impairment. An MMSE score of 27–30, 21–26, 10–20, and 0–9 was defined as normal, mild PND, moderate PND, and severe PND, respectively.

The main outcome measures were MMSE scores before and 24 h after anesthesia. The secondary outcome measures were as follows: blood cell count changes on Days 1, 3, and 6 postoperatively (white blood cell [WBC] count, percentage of lymphocytes [Lym%], and percentage of neutrophils [Neu%]). The safety indicators were adverse reactions that may be associated with the use of Des during the trial, including allergies, itching, rashes, nausea, vomiting, diarrhea, and shock.

### 2.5 MMSE scores

MMSE scores^15^ are closely related to educational level, and the normal threshold is as follows: for illiterate individuals >17, for those who have completed primary school education >20, and for those who have completed junior high school and above >24. The inclusion criterion of this study was an MMSE score > 24. Among the population included in this study, we screened those with a preoperative MMSE score ≥ 24, which indicated that the patients could well understand the contents of the MMSE score, regardless of their educational background. As long as their preoperative MMSE score ≥ 24, they could be included in our study, because the patients with preoperative MMSE scores lower than 24 are difficult to understand the relevant content of MMSE score, which brings great difficulties to postoperative MMSE.

### 2.6 Sample size

PowerAndSampleSize.com was used for sample estimation. It has been reported that the incidence of early PND in elderly patients after noncardiac surgery is 39%, and the estimated incidence of early PND after Dex + Des anesthesia is 15%, significance level (*α*) = 0.05, power (1 – *β*) = 0.80, *N* = (*U*_*a*_ + *U*_*β*_)^2^2*P*(1 − *P*)/(*P*_1_ *− P*_2_)^2^, *n* = 71 ≈ 80, calculated according to the formula, and the sample size of each group is 80 cases.^16^

### 2.7 Statistical analysis

All data were analyzed using SPSS 22.0 version (IBM Corporation). Independent‐samples *t*‐test and the *χ* ^2^ test were used for quantitative and qualitative data, respectively, and the results were expressed as mean ± standard and median (interquartile [range]), respectively. Pearson's correlation was used to correlate PND and inflammation. *p* < 0.05 was considered statistically significant.

## 3 RESULTS

### 3.1 Comparison of the basic information of each group

Table 1 shows the patient characteristics in each group. The male‐to‐female ratio in the Dex + Des group was 31:24, and the ratio in the other group was 27:32; there was no statistical difference between the two groups (*p* = 0.082). The mean age (67 vs. 68 years), mean height (160 vs. 157 cm), and mean weight (60 vs. 56.5 kg) were not statistically significant between the Dex + Des group and the Des group (*p* = 0.262, 0.983, 0.433). A total of 15, 5, 11, 11, 1, and 12 patients in the Dex + Des group underwent laparoscopic cholecystectomy, laparoscopic inguinal hernia repair, urological surgery, internal fixation for fractures, laparotomy, and other types of surgery, respectively. In the Des group, 11, 9, 4, 21, 1, and 13 patients underwent laparoscopic cholecystectomy, laparoscopic inguinal hernia repair, urological surgery, internal fixation for fractures, laparotomy, and other types of surgery, respectively. There was no significant difference in the surgery type between the two groups (*p* = 0.153).

**Table 1:** Demographic data of patients in each group

		Dex + Des group	Des group	*p* Value
Sex (male:female)		31:24	27:32	0.082
Age (years)		67 (60–80)	68 (60–89)	0.262
Height (cm)		160 (145–175)	157 (145–176)	0.983
Weight (kg)		60 (45–80)	56.5 (44–71)	0.433
Education level (%)				0.649
Illiterate		5.4	3.4	
Primary school		56.3	62.7	
Junior secondary school		18.2	18.6	
Senior secondary school		20	15.2	
Coexisting diseases (dropped)				
Anesthesia time (min)		105.73 ± 44.66	107.20 ± 51.78	
Operation time (min)		76.20 ± 38.33	75.55 ± 43.93	
Surgical type				0.153
Laparoscopic cholecystectomy		15	11	
Laparoscopic inguinal hernia repair		5	9	
Urological surgery		11	4	
Internal fixation for fractures		11	21	
Laparotomy		1	1	
Other		12	13	

*Note*: Data are shown as median (IQR [range]) or mean ± standard.

Abbreviations: Des, desflurane; Dex, dexmedetomidine; IQR, interquartile range.

### 3.2 Incidence of PND and comparison of MMSE scores in each group

**Figure 2:**
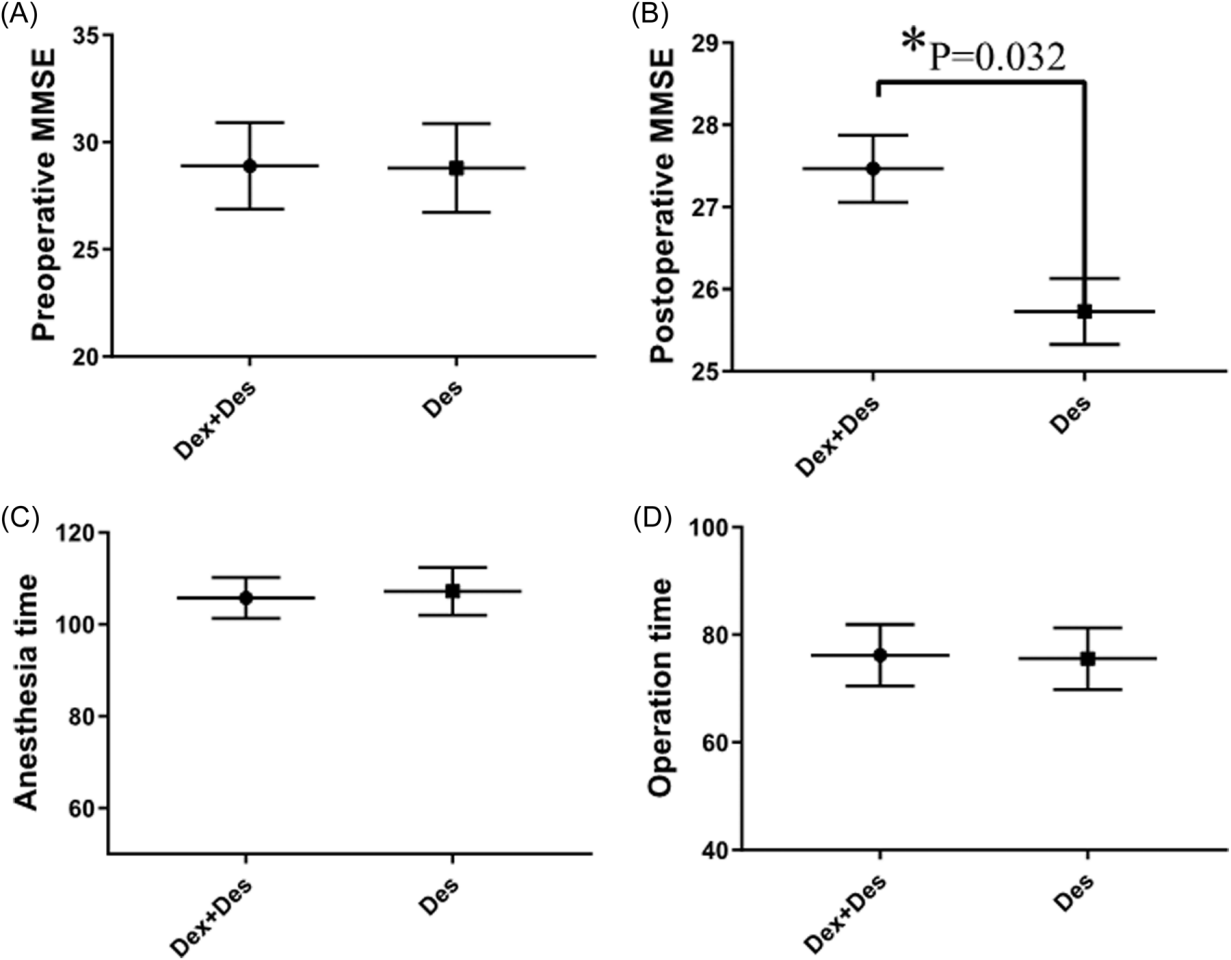
Comparison of MMSE scores, anesthesia time, and operative time between the Dex + Des and Des groups. MMSE scores of each group before (A) and 24 h postoperatively (B), the anesthesia time (C), and the operative time (D) of each group. Dex + Des, combined treatment of dexmedetomidine and desflurane; Des, desflurane; MMSE, mini‐mental state examination. *p < 0.05 compared with Dex + Des group and Des group by using Independent‐samples *t*‐test.

Table 2 shows the analysis of PND in each group. The incidence of PND was 14.5% in the Dex + Des group and 27.1% in the Des group (*p* = 0.100). We found that there was no significant difference in the preoperative MMSE scores for each group (the Dex + Des group vs. the Des group: 28.89 ± 2.01 vs. 28.79 ± 2.07, *p* = 0.150), while the postoperative MMSE scores for the Dex + Des group were significantly higher than those of the Des group (27.24 ± 4.01 vs. 26.64 ± 4.54, *p* = 0.032). There was no significant difference in the operative time (Dex + Des group vs. Des group: 76.20 ± 44.66 vs. 75.56 ± 43.93 min, *p* = 0.937) and the anesthesia time (Dex + Des group vs. Des group: 105.73 ± 2.07 vs. 107.20 ± 51.78 min, *p* = 0.878) (Figure 2A–D).

**Table 2:** Analysis of postoperative PND in each group

	None PND (*n*)	Mild PND (*n*)	Moderate PND (*n*)	Severe PND (*n*)	Occurrence rate (%)
Dex + Des group	47	4	4	0	14.5
Des group	43	10	6	0	27.1
*p* Value					0.1

Abbreviations: Des, desflurane; Dex, dexmedetomidine; PND, postoperative neurocognitive disorder.

### 3.3 Changes in inflammatory cell levels in each group after surgery

**Figure 3:**
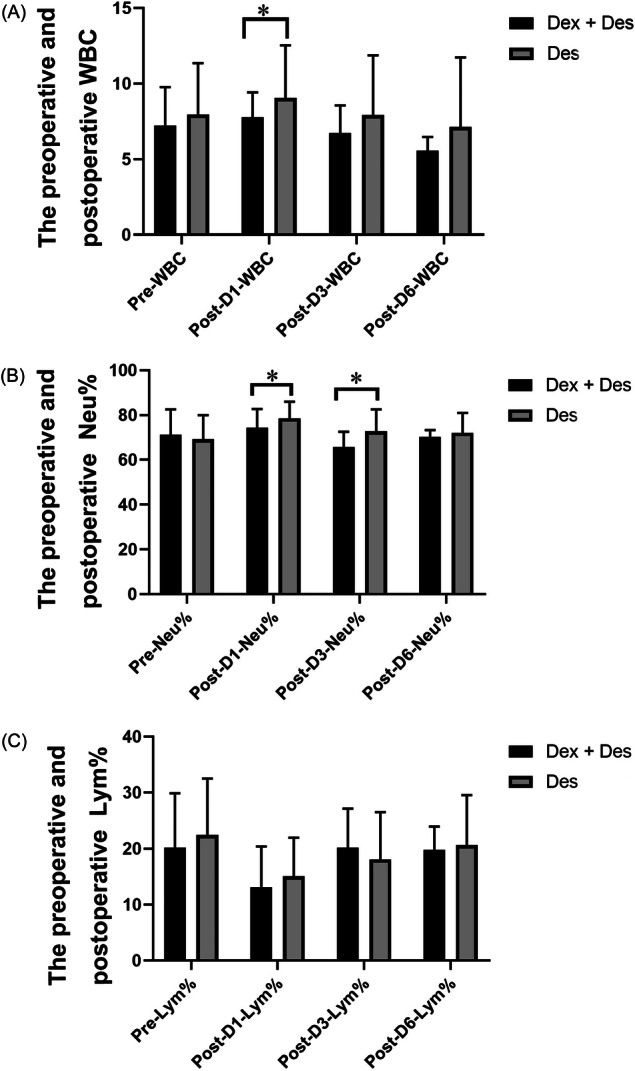
Blood cell counts of each group preopertively and 1, 3, and 6 days after surgery. (A) The preoperative and postoperative WBC (x10^12^). Pre‐WBC, pre‐operative white blood cells; Post‐D1‐WBC, white blood cells on the first day after surgery; Post‐D3‐WBC, white blood cells on the third day after surgery; Post‐D6‐WBC, white blood cells on the sixth day after surgery; (B) The preoperative and postoperative Neu%. Pre‐Neu%, pre‐operative neutrophil percentage; Post‐D1‐Neu%, percentage of neutrophils on the first day after surgery; Post‐D3‐Neu%, percentage of neutrophils on the third day after surgery; Post‐D6‐Neu%, percentage of neutrophils on the sixth day after surgery; (C) The preoperative and postoperative Lym%. Pre‐Lym%, Pre‐operative lymphocyte percentage; Post‐D1‐Lym%, percentage of lymphocytes on the first day after surgery; Post‐D3‐Lym%, percentage of lymphocytes on the third day after surgery; Post‐D6‐Lym%, percentage of lymphocytes on the sixth day after surgery; Lym%, percentage of lymphocytes; Neu%, percentage of neutrophils; WBC, white blood cell; Dex + Des, combined treatment of dexmedetomidine and desflurane; Des, desflurane. *p < 0.05 compared with Dex + Des group and Des group.

We collected and compared the preoperative blood cell counts in each group. Before surgery, the preoperative WBC counts were not statistically significant between the groups (the Dex + Des group vs. the Des group: 7.23 ± 2.54 vs.7.94 ± 3.42, *p* = 0.164; Figure 3A). The Neu% and the Lym% were not significantly different between the groups (the Dex + Des group vs. the Des group: Neu%: 71.16 ± 11.41 vs. 69.24 ± 10.74, *p* = 0.359; Lym%: 20.14 ± 9.73 vs. 22.44 ± 10.07, *p* = 0.214; Figure 3B,C). It is interesting to compare the levels of WBC before and after surgery. We found that the level of WBC decreased significantly in the Dex + Des group on the first day after surgery compared to the Des group (the Dex + Des group vs. the Des group: 7.76 ± 1.66 vs. 9.05 ± 3.48, *p* = 0.02; Figure 3A). The Neu% on the first and third day after surgery was significantly lower in the Dex + Des group than that in the Des group (Dex + Des group vs. Des group: post‐D1‐Neu%: 74.46 ± 8.18 vs.78.52 ± 7.44, *p* = 0. 007; post‐D3‐Neu%: 65.75 ± 6.76 vs. 72.74 ± 9.82, *p* = 0.028; Figure 3B). Therefore, the use of Des and Dex significantly reduced the neutrophil count after surgery. The Lym% was not significantly different postoperatively (the Dex + Des group vs. the Des group: post‐D1‐Lym%: 13.17 ± 7.23 vs. 15.07 ± 6.89, *p* = 0.167; post‐D3‐Lym%: 20.17 ± 6.96 vs. 18.08 ± 8.44, *p* = 0.431; post‐D6‐Lym%: 19.82 ± 4.14 vs. 20.62 ± 8.93, *p* = 0.838; Figure 3C).

### 3.4 Relationship between postoperative inflammatory cell levels and MMSE scores

**Figure 4:**
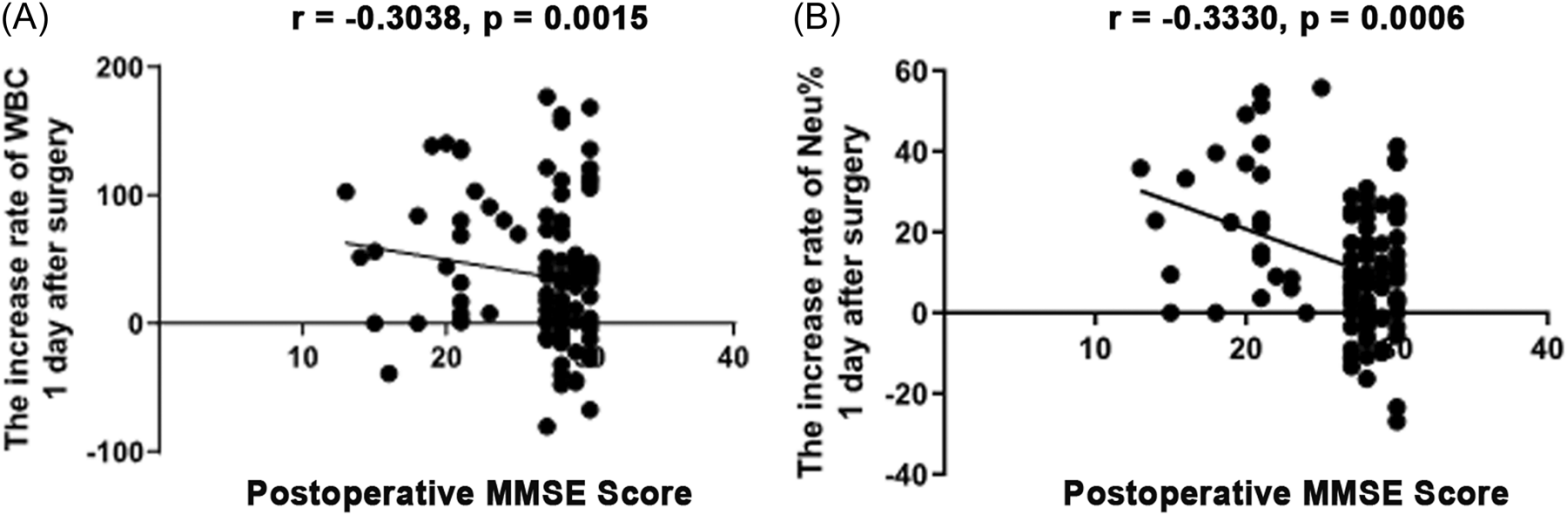
Correlation analysis between MMSE score and the increase rate of post‐operative WBC count and Neu% (A) MMSE score was negatively correlated with the post‐operative increase rate of WBC count (*r* = −0.3038, *p* = 0.0015). (B) MMSE score was negatively correlated with the post‐operative increase rate of Neu% (*r* = ‐0.3330, *p* = 0.0006). MMSE, mini‐mental state examination; Neu%, percentage of neutrophils; WBC, white blood cell.

We used Pearson's correlation analysis to analyze the percentage change of WBC and neutrophils and the MMSE score on the first postoperative day. We found that the percentage of changes in the WBC count on the first postoperative day was negatively correlated with MMSE scores (*r* = −0.3038, *p* = 0.0015), and the percentage of changes in the neutrophil count on the first postoperative day was also negatively correlated with MMSE scores (*r* = −0.3330, *p* = 0.0006). The lower the MMSE score, the more the level of WBC and neutrophils increased. (Figure 4). There was no correlation between the Lym% changes and the MMSE score.

## 4 DISCUSSION

In this study, we found that the postoperative MMSE scores of elderly patients treated with Dex and Des were significantly higher than those treated with Des alone. Compared with Des alone, Dex and Des significantly reduced the percentage of WBCs on a postoperative day 1 and the Neu% on postoperative days 1 and 3 in elderly patients. We further found that the lower the MMSE score in elderly patients, the higher the number of WBCs and neutrophils in their blood after surgery.

POD and postoperative neurocognitive recovery delay are the most common perioperative cognitive disorders that occur after surgical anesthesia.^17^ There are many ways in which patients may develop neurocognitive disorders after admission; surgery and anesthesia are common ways and have different effects for each patient.^18^ An important mechanism that causes neurocognitive disorders during the perioperative period is neuroinflammation, which is characterized by inflammatory imbalance and neuronal damage.^19^ For the patients and their families, perioperative neurologic dysfunction often increases the duration of hospitalization and the cost of hospitalization, brings the huge mental and economic pressure, increases the medical resources and burdens society. Clinical evaluation is also combined with biological indicators; however, there are still many problems that have not been resolved, such as which neuropsychological scales should be used and which cognitive functions should be evaluated, and there are no recognized diagnostic standards. The MMSE scale has been widely used to evaluate the cognitive functions of surgical patients. MMSE^15^, ^20^ is a concise scale with a total of 30 questions covering six topics and a short assessment time of 5–10 min. It is easily accepted by the elderly and is the preferred scale for screening dementia. In this study, an MMSE score < 26 postoperatively indicates cognitive impairment. An MMSE score of 27–30, 21–26, 10–20, and 0–9 was defined as normal, mild PND, moderate PND, and severe PND, respectively. In the Dex + Des group, 4, 4, and 0 patients, respectively, had mild PND, moderate PND, and severe PND, but in the Des group, 10, 6, and 0 patients had mild PND, moderate PND, and severe PND, respectively. This means that the use of Des could decrease the occurrence of mild PND. The limitations of the Montreal Cognitive Assessment (MoCA) scale are mainly reflected in the floor effect caused by the complexity of the topics compared with MMSE. Because the language and culture of China are quite different from that of foreign countries, it is quite difficult to apply MoCA. In recent years, research on neurocognitive disorders during the perioperative period has made some progress. Cognitive evaluation has gradually expanded from postoperatively to preoperatively. In the current study, we used the MMSE scale to evaluate the cognitive function in patients with complications following surgery and anesthesia.

### 4.1 General anesthesia using Des combined with Dex can reduce the occurrence of PND in elderly patients

In our study, we found that general anesthesia using Des combined with Dex can reduce the occurrence of PND in elderly patients. Studies in critically ill patients have confirmed that Dex can reduce the incidence of PND in patients.^21^, ^22^, ^23^ A meta‐analysis in 2021 of 15 randomized control study, contains a total of 2183 patients, including 1079 cases of patients who used the Dex, 1104 cases did not receive Dex, the results showed whether the inhalation anesthesia or systemic intravenous anesthesia in adult patients undergoing heart surgery, Dex administration reduced the likelihood of postoperative cognitive and behavioral dysfunction by at least 43%.^24^ Des has been found to affect Moto neuron viability and maturation in vitro, but the α2‐adrenergic receptor agonist Dex attenuates the effects of Des on motor neurons, and this process is mediated by nuclear factor‐κB (NF‐κB) signaling.^10^ In pediatric patients undergoing tonsillectomy or adenoidectomy, maintenance of anesthesia with Des and Dex can effectively prevent emergence agitation.^25^ Although anesthesia with Des provides rapid recovery, a higher incidence of severe recovery agitation (up to 80%) has been found in pediatric patients after anesthesia with Des.^26^ In children under general anesthesia, a continuous infusion of low‐dose Dex without a loading dose did not cause hemodynamic changes, but reduced the incidence of delirium after anesthesia with Des in children undergoing strabismus surgery.^27^ [Correction added on 17 May 2024, after first online publication: The word “postoperative POD” was revised to “PND” in this section.]

### 4.2 Dex reduces PND

A recent study reported that the risk of PND after inhalation anesthesia is higher than of intravenous anesthesia, with an odds ratio of 0.52.^28^ Surgery such as source of stress can lead to the non‐specific response of the body, also known as the stress response, which is manifested as the sympathetic nervous excitement, increased pituitary and adrenal cortical hormone secretion, and increased blood glucose, blood pressure, HR and breathing rate. Intraoperative excessive stress reaction can lead to fluctuations in perioperative vital signs, ultimately lead to cognitive dysfunction.^29^, ^30^ Des is a methyl ether structure of inhalation anesthetics, and it has been reported that it can induce lung parenchymal inflammation and lipid peroxidation, increase alveolar macrophages, and cause per bronchial infiltration and edema in rats.^31^ Compared with the use of propofol alone, the level of postoperative inflammation is higher on anesthesia with Des.^32^ Dex reduces the level of postoperative inflammatory factor expression in patients via the PI3K‐Akt signaling pathway, thereby accelerating postoperative cognitive recovery.^33^ Animal studies in elderly mice have shown that pretreatment with Dex may reduce neuroinflammation and the incidence of PND in elderly mice by inhibition of the hippocampal TLR4 NF‐κB signaling pathway.^34^ In a study of sevoflurane (SEV)‐induced PND in anesthetized rats, we found that Dex improved SEV‐induced POCD by increasing Mir‐129 and inhibiting the phosphorylation of TLR4 and NF‐κB P65.^35^ In neurosurgery patients with intracranial aneurysm open‐brain surgery, the application of Dex can protect the patient's brain, minimizing the operation's influence on hemodynamics, relieve postoperative pain.^36^ In primary cultured neurons, we found that Dex attenuated the neurotoxicity of propofol on hippocampal neurons.^37^ In our trial, we found that after using Dex, the ratio of inflammatory cells, such as the neutrophil and WBC counts on the first postoperative day, was lower than that before surgery. This finding is consistent with previous reports. The use of Dex combined with Des can reduce postoperative inflammatory cell levels to a greater extent, which indicates that the use of Dex combined with Des is beneficial for patient rehabilitation. We also found a negative correlation between the postoperative MMSE score and the inflammatory cell count, which indirectly indicates that a reduction in the postoperative inflammatory response reflects the accelerated recovery of postoperative cognitive function. [Correction added on 17 May 2024, after first online publication: The word “postoperative POD” and “POCD” was revised to “PND” in this section.]

## 5 LIMITATIONS

Because we only determined the MMSE score 24 h postoperatively, we did not analyze Pearson's correlation coefficients on the third and sixth day postoperatively. In our subsequent study, we will use a variety of scales to evaluate the awareness and cognitive level of patients. We will not only determine the MMSE score at 24 h after surgery but also the MMSE score and the MoCA score at 3 days, 7 days, and 1 month after surgery, as well as the corresponding blood inflammatory cell level. The MoCA score is more useful for the assessment of mild cognitive impairment than the MMSE score, because it covers more extensive and comprehensive cognitive fields, and the score distribution is more reasonable.

In this study, we finally included 55 patients in the Dex + Des group and 59 patients in the Des group; these were lower than the sample size estimated by us (80 cases). We used G*power (version 3.1.9.2) software to estimate the sample size, and it was estimated that the occurrence of PND in the Dex + Des group was 50% lower than that in the Des group, and the sample size in each group was 51 patients. After the completion of the test, the incidence of PND in the Dex + Des group was 14.5% and that in the Des group was 27.1%. The incidence of PND in the Dex + Des group was 46% lower than that in the Des group. Power (1 − β) = 0.78 ≈ 0.8 was obtained by sample size verification using Gpower software, so the sample size in our study was representative to some extent.

## 6 CONCLUSION

The use of Dex combined with Des reduced the incidence of PND, which reduced the postoperative inflammatory cell count, especially on the first and third postoperative days. This indicates that the use of Dex or Des significantly reduced the neutrophil ratio after surgery, and indirectly indicates that Dex or Des did not significantly reduce the lymphocyte ratio after surgery. Clinical anesthesia, when combined with Dex, reduces the incidence of PND, reduces the length of hospital stay for patients, reduces the cost of hospitalization, and saves social medical resources, and is worth promoting.

## AUTHOR CONTRIBUTIONS

All authors are responsible for the accuracy of data in this study and approved the final version of the manuscript. Yue Hu, Yong Ye, and Hui Lin designed and supervised the whole study; Jun‐Jie Chen, Ting‐Ting Sun, Gong‐Wei Zhang, Na Wang, and Yuan‐Hang Shu performed all the experiments; Xue Gong, Fei‐Fei Ran, and Jia‐Li Zhang contributed to the data collection and analysis; and Yong Tao contributed toward guidance of experiments and data verification.

## CONFLICT OF INTEREST

The authors declare no conflict of interest.

## ETHICS STATEMENT

This study was a randomized, parallel controlled trial, double‐blind trail. The current study complied with the Consolidated Standards of Reporting Trials (CONSORT) guidelines, was conducted between May 2019 and February 2020, and was approved by the Ethics Committee of The First People's Hospital of Shuangliu District, Chengdu, (Approval number: 20180101). Similarly, this study was approved by the Chinese Clinical Trial Registry (Clinical Trial Registration Number: ChiCTR1900023385). Informed consent was obtained from all the patients.

## Data Availability

All data generated or analyzed during this study are included in this published article.
